# Analysis of the pharmacological mechanism of Banxia Xiexin decoction in treating depression and ulcerative colitis based on a biological network module

**DOI:** 10.1186/s12906-020-02988-3

**Published:** 2020-06-29

**Authors:** Ying Yu, Gong Zhang, Tao Han, Hailiang Huang

**Affiliations:** 1 College of Traditional Chinese Medicine, Shandong University of Traditional Chinese Medicine, Jinan, 250355 China; 2 College of Traditional Chinese Medicine, Shandong University of Traditional Chinese Medicine, Jinan, 250355 China; 3 Graduate Office of Shandong University of Traditional Chinese Medicine, Jinan, 250355 China; 4 College of Rehabilitation Medicine, Shandong University of Traditional Chinese Medicine, Jinan, 250355 China

**Keywords:** Banxia Xiexin decoction, Depression, Ulcerative colitis, Network pharmacology, Signal pathway, Target, Module analysis

## Abstract

**Background:**

The network pharmacology method was used to predict the active components of Banxia Xiexin decoction, its targets and the key signalling pathways that are activated in the treatment of depression and ulcerative colitis to explore the common mechanism.

**Methods:**

The active components and targets of Banxia Xiexin decoction were obtained by searching the ETCM,TCMSP and TCMIP database. The disease targets of depression and ulcerative colitis were obtained by combining the following the DisGeNET, OMIM,Drugbank,CTD and PharmGKB disease databases. The drug and disease target genes were obtained from the intersection of the herbal medicine targets and the disease targets and were imported into the STRING platform for the analysis of PPI network. The network modules were constructed using Cytoscape software. An analysis of the functional annotations of GO terms and KEGG signalling pathways was performed for each network module. Then, the tissue distribution, sub-cellular distribution and protein attributes of the key targets in the pathway were analysed by the BioGPS, Genecards and DisGeNET databases.

**Results:**

The mechanism of Banxia Xiexin Decoction in the treatment of depression and ulcerative colitis is related to drug reaction, steroid metabolism, lipid metabolism, inflammatory response, oxidative stress response, cell response to lipopolysaccharide, insulin secretion regulation, estradiol response and other biological functions, mainly through the regulation of 5-hydroxytryptamine synaptic, arachidonic acid metabolism, HIF-1 signaling pathway and NF-kappa B signaling pathway can achieve the effect of same treatment for different diseases.

**Conclusions:**

The mechanism of Banxia Xiexin Decoction in treating different diseases involves direct or indirect correlation of multiple signal pathways, mainly involved in drug metabolism and lipid metabolism, but also through comprehensive intervention of the body’s nervous system, immune system, digestive system and other systems. The effective components of Banxia Xiexin Decoction are mainly act on eight key target proteins (such as ALB, IL6, VEGFA, TNF, PTGS2, MAPK1, STAT3, EGFR) to carry out multi-target effect mechanism, biological mechanism of treating different diseases with the same treatment, and related mechanism of overall treatment, which provide theoretical reference for further research on the material basis and mechanism of Banxiaxiexin decoction on antidepressant and prevention and treatment of ulcerative colitis.

## Background

Depression is a chronic and recurrent mental disorder. The main clinical characteristics of patients are marked and lasting depression, accompanied by physical symptoms of gastrointestinal diseases, which can impair social functions and lead to suicidal tendencies. Thus, depression is very harmful to the physical and mental health of patients [1–3]. Ulcerative colitis is a chronic gastrointestinal disease caused by a variety of pathogenic factors. Ulcerative colitis is mainly characterized by abdominal pain, diarrhoea, mucopurulent bloody stool and other symptoms. In severe cases, ulcerative colitis can be complicated with an intestinal abscess, intestinal perforation and colorectal cancer [4–6]. Therefore, the two diseases have the characteristics of a complicated aetiology, diverse syndromes, and a long course. The occurrence and development of the diseases also have the potential characteristics of mutual influences and correlation, that is, patients with depression often exhibit gastrointestinal dysfunction that inhibits the internal vitality of patients, reduces gastrointestinal motility and promotes the occurrence of ulcers. Patients with severe depression may also develop intestinal cancer. In addition, the emotional symptoms of depression will also alter the regulatory functions of the related gastrointestinal nerves and motor activity to produce symptoms, such as a loss of appetite, constipation, abdominal distention, and diarrhoea. However, these gastrointestinal symptoms will also aggravate the anxiety and depression of patients, and the gastrointestinal dysfunction experienced by patients with chronic recurrent inflammatory bowel disease will easily cause anxiety and depression. These unpleasant emotions will also affect ulcer healing and disease recovery, and even lead to a vicious cycle of aggravating disease-related damage [7–9].

With the rapid development of modern society and the economy, the accelerated pace of life, and the intense pressure of competition, the number of patients with the two diseases has shown an increasing trend, which not only seriously distress the normal life and work of patients but also imposes a heavy burden on the family and society. Currently, Standard treatment often uses an aminosalicylic acid preparation, corticosteroid hormones, and immunosuppressants as the main treatment, combined with antidepressants. Although these drugs display short-term clinical efficacy, different degrees of side effects occur with the prolongation of the treatment, which will seriously reduce the compliance of patients with the treatment process and the curative effect of the disease. Traditional Chinese medicine has the unique advantages of overall regulation, safety and effectiveness in the treatment of depression and ulcerative colitis. Traditional Chinese medicine not only has a profound foundation in clinical practice but also has led to the development of a large number of classic prescriptions for treatment. Thus, traditional Chinese medicine has gradually become more widely used. Banxia Xiexin decoction, which was first published in the Treatise on Cold Damage and Miscellaneous Disease, is composed of seven herbs, namely, Banxia, Ganjiang, Scutellaria, Coptis, ginseng, liquorice and jujube. This prescription can effectively improve gastrointestinal inflammation, regulate gastrointestinal motility and anti-ulcer effectively in clinical [10–12]. Related animal experimental studies show that this formula can effectively increase CD^8+^ and regulate CD^4 +^ / CD^8 +^ balance in UC model rats [13]. It can regulate the secretion and movement of gastrointestinal tract, repair the barrier of gastrointestinal mucosa, reduce the infiltration of inflammatory cells in mucosa and promote the healing of ulcer [14]. Relevant research has confirmed that the pathogenesis of ulcerative colitis is closely related to environmental, mental, psychological and other factors. The model of ulcerative colitis in rats induced by TNBS+ ethanol was established to study the therapeutic effect and mechanism of Banxia Xiexin Decoction on ulcerative colitis. The degree of ulcer damage, ulcer area, wet weight of intestine and MPO activity were measured. The anxiety behavior of rats was also evaluated by the elevated cross labyrinth method. The results showed that Banxia Xiexin Decoction had anti-inflammatory and anti anxiety effects [15]. At the same time, the prescription has a good clinical effect on the patients with depression accompanied by gastrointestinal symptoms such as fullness of epigastric distention, less sodium, loose stools and so on [16, 17]. According to recent clinical studies have shown that the traditional Chinese medicine Banxia Xiexin Decoction can significantly reduce the scores of Hamilton Depression Scale and self rating depression scale of depression patients. In addition, animal experimental studies have also confirmed that this traditional Chinese medicine can effectively improve the behavior and activity state of liver depression model rats, and can effectively shorten the duration of immobility state in tail suspension test and forced swimming test of mice It shows that the Jingfang has a significant antidepressant effect [18, 19].

Based on previous clinical experience, modern clinical practice and animal experiments using traditional Chinese medicine, Banxia Xiexin decoction effectively displays the comprehensive advantages of treating different diseases, treating the viscera and treating specimens with the same formulation through component coordination, target coordination and efficacy coordination among various herbs. However, there is a lack of comprehensive and systematic understanding on the material basis and molecular mechanism of its efficacy, and there is no study on the biological mechanism of Banxia Xiexin decoction in treating depression and ulcerative colitis with different diseases. Therefore,this study aims to screen the active components and predicted targets of the herbs contained in Banxia Xiexin decoction by using the biological module analysis of network pharmacology to better understand the common targets, molecular mechanism, tissue localization and subcellular distribution of the traditional Chinese medicine in the treatment of different diseases. In addition, the relevant networks and biological functions are analysed to explore the multi-target mechanism, the biological mechanism of simultaneously treating different diseases, and the related mechanism of the overall treatment with the classical prescriptions in a comprehensive, multi-level and systematic manner. We aim to provide a reliable reference value for subsequent experimental verification, clinical application, and new drug research and development.

## Methods

### Screening of the active components and prediction of the targets of Banxia Xiexin decoction

Banxia Xiexin decoction contains seven Chinese herbal medicines: Banxia (Arum Ternatum Thunb), Scutellaria (Scutellaria baicalensis Georgi), Coptis (Coptis chinensis Franch), ginseng (*Panax ginseng* C. A. Mey), ginger (*Zingiber officinale* Rosc), jujube (*Ziziphus jujuba* Mill) and liquorice (Glycyrrhiza uralensis Fisch). The active components and targets of Banxia Xiexin decoction were obtained by searching the Encyclopedia of Traditional Chinese Medicine (ETCM,http://www.nrc.ac.cn:9090/ETCM/) [20, 21], this platform combines a variety of authoritative algorithms, including physical and chemical properties, drug-forming evaluations, absorption and distribution of metabolic excretion index scores, target prediction, etc. Furthermore, the high correlation target of a drug-compound-target is a QED score greater than 0.8. The active chemical components and predicted target of each herb contained in Banxia Xiexin decoction were collected mainly according to the similarity of the chemical fingerprint of the traditional Chinese medicine components and the known drugs. Then, add the TCMSP (http://lsp.nwu.edu.cn/tcmsp.php) database was used to search all the chemical composition information of the single Chinese medicine. According to the recommended screening threshold conditions of the platform, when OB ≥ 30% and DL ≥ 0.18, get the key active ingredients of the Chinese medicine were obtained [22]. The candidate active ingredients were input to PubChem (https://pubchem.ncbi.nlm.nih.gov/) database to search and download the molecular structure formula and PubChem CID of the corresponding chemical composition, and then select the corresponding target of the compound whose species is “*Homo sapiens*” in SwissTargetPrediction (http://swisstargetprediction.ch/) platform according to the chemical similarity between the active compound ligands. Seven herbs were added to TCMIP (http://www.tcmip.cn/) database to establish the chemical composition database of Banxia Xiexin Decoction. According to the target prediction interface of traditional Chinese medicine, all chemical composition prediction targets with drug similarity score ≥ 0.8 were selected [23–25]. The obtained active components of herbal medicine were analysed by combining the data from the relevant literature. The UniProtkB search function in the UniProt (http://www.uniprot.org/) database was used to input the names of the predicted target proteins, and the species was set to *Homo sapiens*. Then, the target protein of each herbal medicine was corrected to the official symbol.

### Assessment of the disease targets for depression and ulcerative colitis

Using the disease databases gene disease association database (DisGeNET), comprehensive human Mendelian genetics database (OMIM), drug target database (Drugbank), comparative toxicogenomic database (CTD), and pharmacogenetics and pharmacogenomics database (PharmGKB), we input the disease gene keywords for depressive disorder, depression and ulcerative colitis. The target genes of the two diseases were searched and screened, and the target genes of each disease database were combined to eliminate the repeated disease targets. Finally, the known disease targets of depression and ulcerative colitis were obtained.

### Constructing the database of intersecting drug-disease target genes

The herbal medicine targets in Banxia Xiexin decoction and the disease targets of depression and ulcerative colitis were separately imported into the OmicShare online tool to generate a Venn diagram. The target genes of Banxia Xiexin decoction for treating depression and ulcerative colitis were mapped, and the target gene database of compounds for treating depression and ulcerative colitis was constructed by extracting the intersecting target genes. The disease target genes that were not related to the components of Banxia Xiexin decoction were removed from the table, and the components that did not match the components of depression and ulcerative colitis were removed. Finally, the disease target genes of the herbs were obtained, and a common target database was established.

### Construction of the protein interaction network

The PPI was constructed and clarified using the STRING network database platform to further elucidate the interactions of the common target genes in the treatment of depression and ulcerative colitis with Banxia xiexin decoction. The protein species was set to “*Homo sapiens*”, and the minimum interaction threshold was set to “Medium Confidence”. The data for the protein-protein interactions were obtained, listed in columns, and saved in. TSV format, and the PPI network was visualized by constructing a graph.

### Construction of the network modules and analysis of the module function annotations

As a closely connected functional group, the biological network module performs the corresponding functions through the interactions of multiple targets in each network module. The data presented in columns of the TSV file were imported into the Cytoscape software, and the key network modules were constructed by using the MCODE Cluster analysis plug-in in the toolbar Apps. By using the online Database for Annotation, Visualization and Integrated Discovery (DAVID6.8), the target genes in the key network modules were analysed by determining the related GO biological processes and signalling pathways that were enriched in the KEGG. The DAVID database integrates various database resources and uses an improved fisher to accurately assess and calculate the results. The *P*-value of each function and pathway was obtained by the method and was corrected using the Benjamini-Hochberg algorithm. The biological signaling pathways with *P* < 0.05 were regarded as statistically significant. The results were sorted according to the P-value, and the top three results were retained.

### Tissue distribution, protein attributes and sub-cellular distribution of the targets in the key pathways

The key targets in the selected signalling pathways were imported into the STRING database to obtain the protein interaction relationship, and the protein interaction network was drawn with the Cytoscape software. According to the Degree value, the key targets were selected and imported into the BioGPS (http://biogps.org), Genecards (http://www.genecards.org/) and DisGeNET (http://www.disenet.org) databases to obtain the tissue distribution, subcellular distribution and protein classification information, respectively.

## Results

### Active components and target selection of Banxia Xiexin decoction

Through searching the ETCM,TCMSP and TCMIP online databases to obtain the compounds and prediction targets of Banxia Xiexin Decoction, and combining the compound information of each database, 364 active compounds were obtained (Including 28 Banxia, 60 Scutellaria, 14 Coptis, 116 ginseng, 7 ginger, 59 jujube, 80 Glycyrrhiza). Using the related target prediction technology in each software to screen the prediction targets of the above-mentioned active ingredients, eliminate the repeated targets, and finally obtain a total of 1551 herbal prediction targets.

### Screening disease targets for depression and ulcerative colitis

**Fig. 1:**
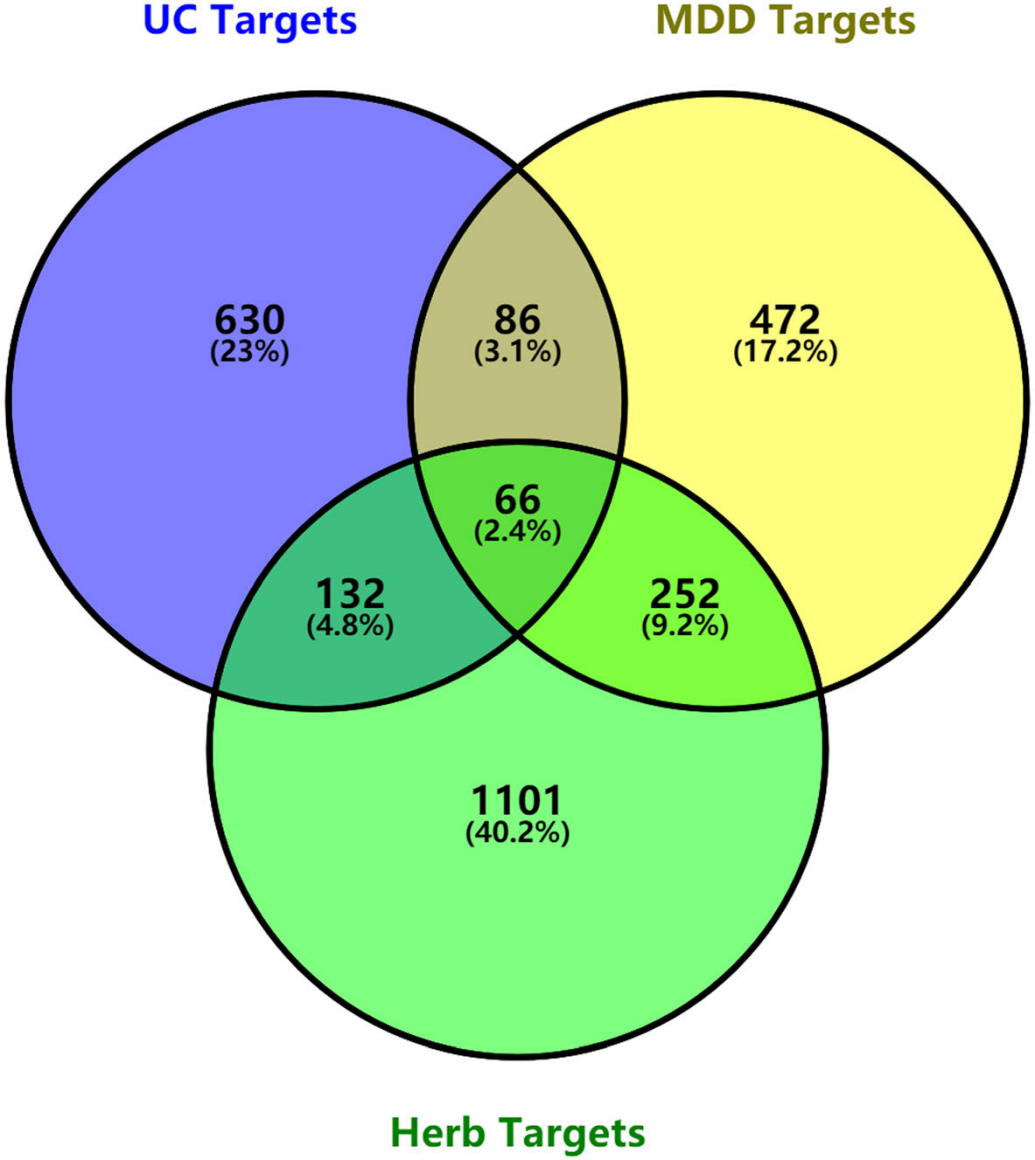
Venn diagram of drug-disease intersection target of Banxia Xiexin decoction for depression and ulcerative colitis

We searched the DisGeNET, OMIM, Drugbank, CTD and PharmGKB databases and screened the known target genes related to depression and ulcerative colitis. We combined the target genes from each database, deleted the duplicate disease targets, and retrieved 876 disease targets for depression and 914 disease targets for ulcerative colitis. The intersection of 1551 predicted herbal medicine targets and two disease targets was obtained to obtain 318 therapeutic targets of Banxia Xiexin decoction for depression and 198 therapeutic targets for ulcerative colitis. Then, 318 depression targets and 198 ulcerative colitis targets were imported into the OmicShare online tool to extract the intersecting target genes and construct a Venn diagram, as shown in Fig. 1. Finally, 66 common targets of Banxia Xiexin decoction in the treatment of depression and ulcerative colitis were obtained.

### Constructing the PPI network of shared targets

**Fig. 2:**
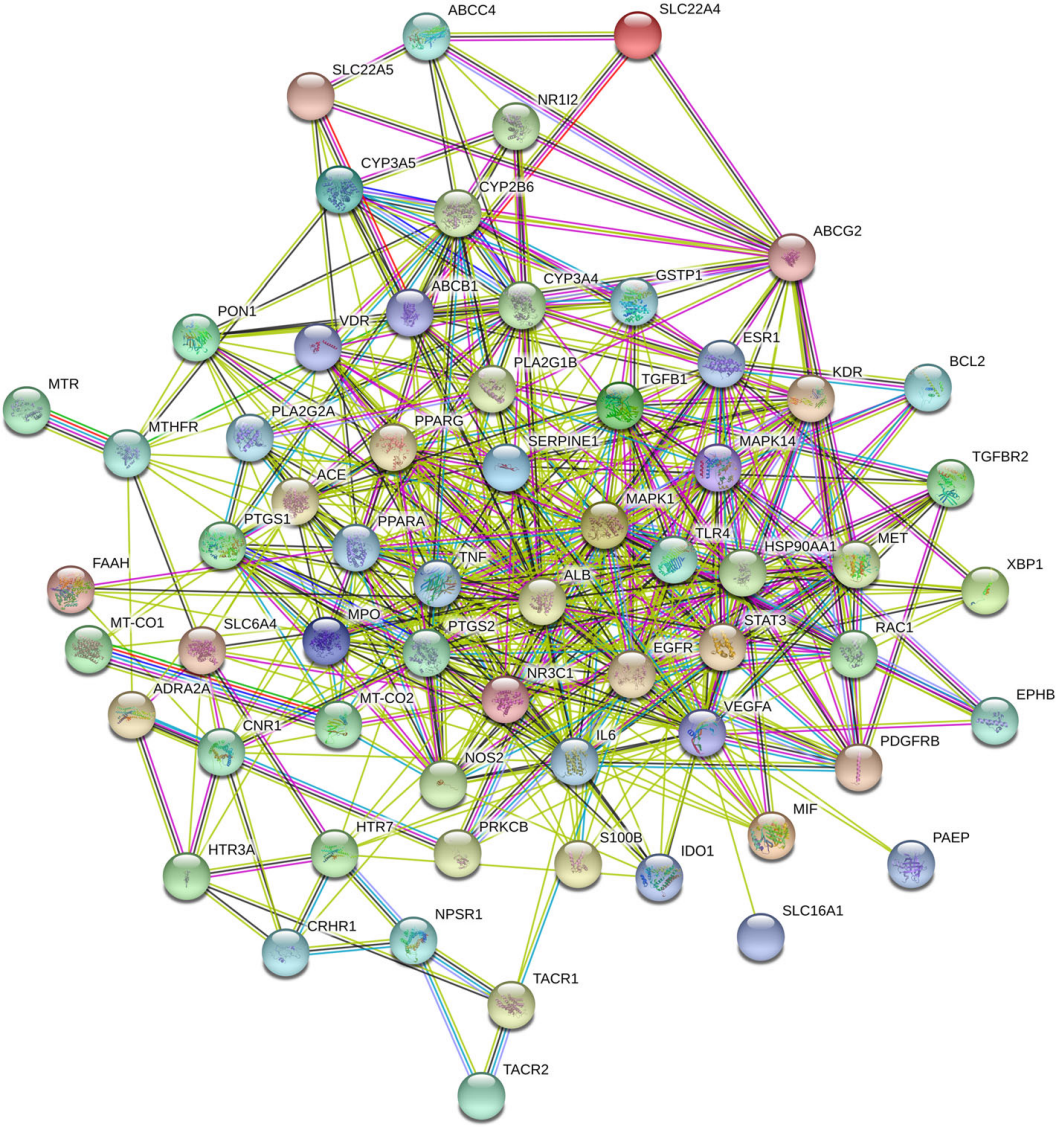
PPI Network Diagram of Common Targets

The 66 intersecting target genes listed above were imported into the STRING software platform to obtain the data columns of protein-protein interactions, construct a PPI network diagram, and better understand the potential mechanism of Banxia xiexin decoction in the treatment of depression and ulcerative colitis. The network contains 65 protein-protein interaction targets and 464 edges that represent protein-protein interactions. The average node Degree value in the network is 14.30, and the average centre clustering coefficient is 0.614, as shown in Fig. 2.

### Identification and analysis of network modules

**Fig. 3:**
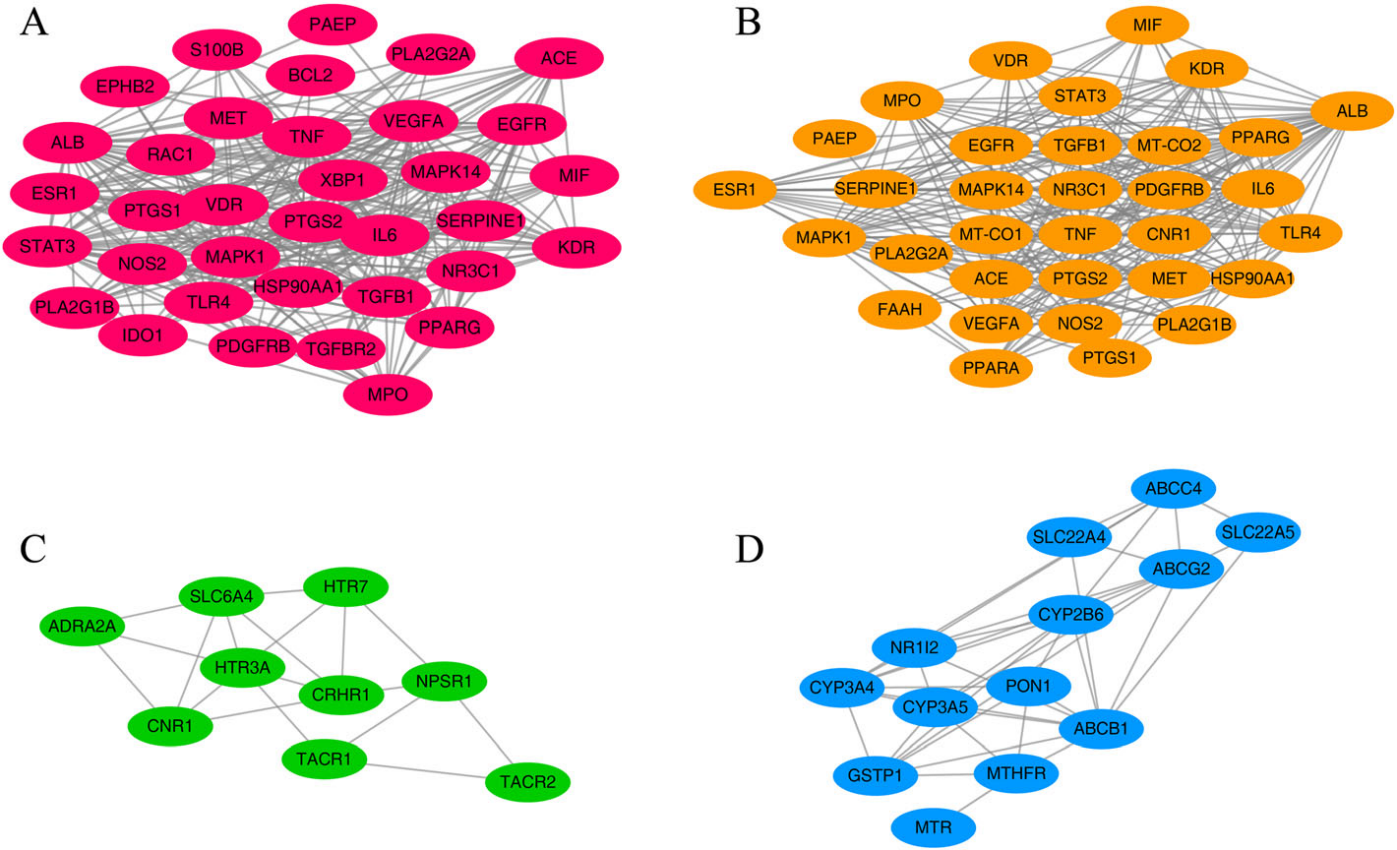
Network module analysis of Banxia Xiexin Decoction in the treatment of depression and ulcerative colitis

The protein interaction data listed above were imported into the Cytoscape software, and the protein interaction network of Banxia Xiexin decoction in the treatment of depression and ulcerative colitis was divided into modules by using the MCODE Cluster plug-in tool, involving 63 nodes and 464 edges. Four key network modules and 90 targets were finally obtained, among which the largest network module was composed of 35 target nodes. The nodal target proteins contained in these modules may play important roles in the treatment of depression and ulcerative colitis with Banxia Xiexin decoction, as shown in Fig. 3. The targets contained in each network module were imported into the DAVID tool for an enrichment analysis of GO biological processes and KEGG pathways. Table 1 lists the top three biological processes and signalling pathways of each network module according to the significance of the *P*-values obtained from the enrichment analysis. Module A was mainly related to the positive regulation of biosynthesis, including pathway in cancer, proteoglycan signaling pathway in cancer and arachidonic acid metabolism pathway. Module B was mainly related to metabolism pathway, including HIF-1 signaling pathway, arachidonic acid metabolism pathway and NF-kappa B signaling Pathway. Module C was mainly related to plasma membrane regulation, mainly involving neuroactive ligand-receptor interaction signaling pathway, serotonergic synapse and calcium signaling pathway. Module D was related to related metabolic processes, mainly involving drug metabolism-cytochrome P450, metabolism of xenobiotics by cytochrome P450 and retinol metabolism signaling pathway.

**Table 1:** Results of enriched GO biological process and KEGG signal pathway analysis results of for module A ~ D (only top three are reserved)

**Module**	**GO Function**	***P-*Value**	**KEGG Signaling Pathway**	***P-*Value**
A	GO:0045944 ~ positive regulation of transcription from RNA polymerase II promoter	1.84E-10	hsa05200:Pathways in cancer	3.42E-10
	GO:0045429 ~ positive regulation of nitric oxide biosynthetic process	2.50E-10	hsa05205:Proteoglycans in cancer	4.86E-10
	GO:0008284 ~ positive regulation of cell proliferation	1.07E-09	hsa00590:Arachidonic acid metabolism	6.48E-03
B	GO:0006367 ~ transcription initiation from RNA polymerase II promoter	1.92E-11	hsa04066:HIF-1 signaling pathway	7.71E-08
	GO:0045429 ~ positive regulation of nitric oxide biosynthetic process	1.11E-10	hsa00590:Arachidonic acid metabolism	1.86E-03
	GO:0048146 ~ positive regulation of fibroblast proliferation	3.81E-08	hsa04064:NF-kappa B signaling pathway	4.83E-02
C	GO:0005887 ~ integral component of plasma membrane	1.25E-07	hsa04080:Neuroactive ligand-receptor interaction	2.01E-06
	GO:0005886 ~ plasma membrane	6.80E-06	hsa04726:Serotonergic synapse	5.14E-03
	GO:0007268 ~ chemical synaptic transmission	1.53E-04	hsa04020:Calcium signaling pathway	1.30E-02
D	GO:0006805 ~ xenobiotic metabolic process	2.07E-07	hsa00982:Drug metabolism - cytochrome P450	1.06E-04
	GO:0008202 ~ steroid metabolic process	3.39E-06	hsa00980:Metabolism of xenobiotics by cytochrome P450	1.36E-04
	GO:0042908 ~ xenobiotic transport	9.81E-06	hsa00830:Retinol metabolism	3.65E-03

### GO biological processes of common target genes and KEGG pathway analysis

**Fig. 4:**
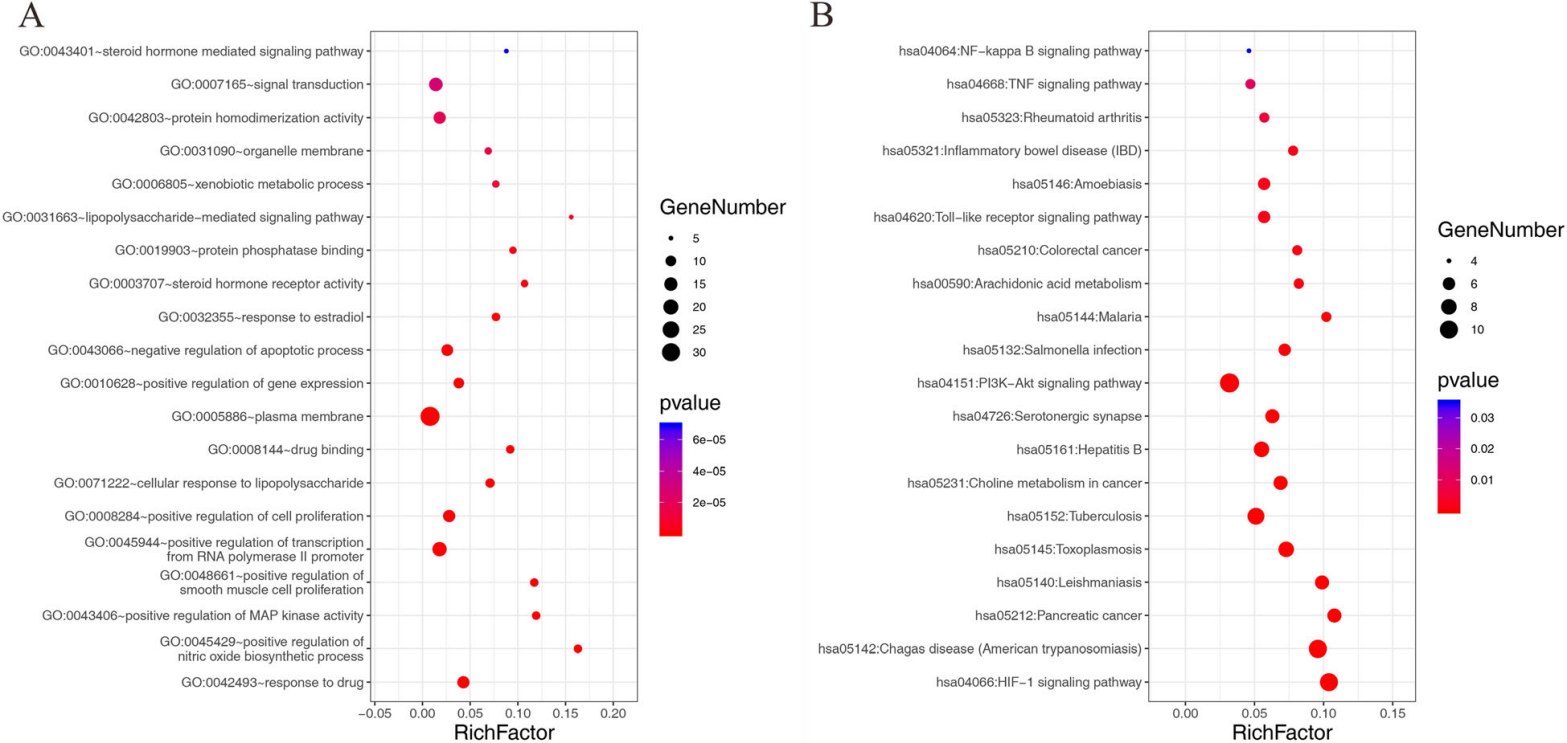
Results of the analysis of enriched GO biological processes (**a**) and KEGG pathways (**b**) of common targets of Banxia Xiexin decoction in the treatment of depression and ulcerative colitis

The 66 common target genes screened above were mapped for an enrichment analysis of the GO terms and KEGG pathways and displayed in the chart shown in Fig. 4. Go biological process analysis showed that drug response, steroid metabolism, lipid metabolism, inflammatory response, oxidative stress response, cell response to lipopolysaccharide, insulin secretion regulation, estradiol response and other processes play an important role in the biological process of Banxiaxiexin Decoction in the treatment of depression and ulcerative colitis. KEGG signaling pathway analysis showed that 5-hydroxytryptamine synapse signaling pathway, arachidonic acid metabolism signaling pathway, HIF-1 signaling pathway, NF-kappa B signaling pathway all played a significant role in the treatment of different diseases.

### Screening targets in key pathways

The targets in the four key signaling pathways selected above were imported into the STRING platform, and the columns of protein-protein interaction data were obtained. The data columns were imported into the Cytoscape software for a network topology analysis. The targets were sorted according to the node degree value, and the frequency of the targets in each pathway was counted. Finally, the key targets with a value higher than the degree median value and the highest frequency were selected. Eight key targets meet these criteria, as shown in Table 2. The same target might be involved in the treatment of different diseases by regulating different signaling pathways. Therefore, to a certain extent, the classic Banxia Xiexin decoction potentially plays a role in regulating different diseases and treating them simultaneously, and these key targets play important roles in the treatment of two different diseases.

**Table 2:** Eight key targets of Banxia Xiexin decoction in common pathways involved in the treatment of depression and ulcerative colitis

**Gene Symbol**	**Uniprot ID**	**Target protein name**	**Gene Symbol**	**Uniprot ID**	**Target protein name**
ALB	P02768	Serum albumin	PTGS2	P35354	Prostaglandin G/H synthase 2
IL6	P05231	Interleukin-6	MAPK1	P28482	Mitogen-activated protein kinase 1
VEGFA	P15692	Vascular endothelial growth factor A	STAT3	P40763	Signal transducer and activator of transcription 3
TNF	P01375	Tumor necrosis factor	EGFR	P00533	Epidermal growth factor receptor

### Tissue distribution, subcellular location and protein attributes of targets in key pathways

**Fig. 5:**
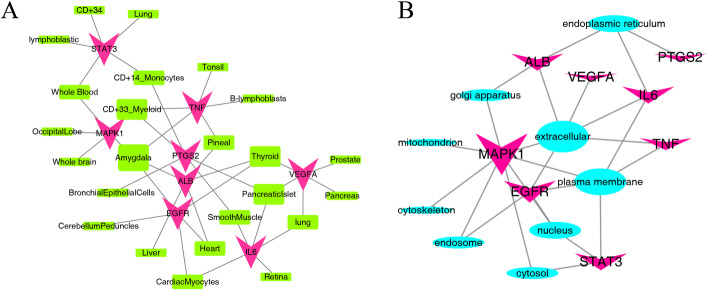
Target-tissue network (**a**) and target-subcellular network (**b**) of eight key targets in common pathways regulated by Banxia xiexin decoction in the treatment of depression and ulcerative colitis

The eight key targets described above were imported into the BioGPS platform to obtain the relevant tissue distribution information. Obtain the tissue information with correlation cutoff value above 0.9 and each gene expression ranking in the top five to make a data list. The obtained data list is imported into the Cytoscape software to draw the “tissue-target”network diagram, as shown in Fig. 5 (A). The red V-shaped triangle node represents the key target, and the green square node represents the tissue. The tissue distribution with highest frequence was amygdala (Degree = 4), pancreaticislet (Degree = 3), lung (Degree = 2) play important roles in the treatment of depression and ulcerative colitis with the Banxia Xiexin decoction, which provides some guidance for the next group of animal experiments. The results also revealed that CD33+ myeloid cells (Degree = 3), CD14+ monocytes (Degree = 2), cardiac myocytes (Degree = 2), B-lymphoblasts (Degree = 1), bronchial epithelial cells (Degree = 1) and other cells also play an important role.

Import the key targets into the Genecards database to obtain the subcellular distribution information. The subcellular location of Confidence = 5 of the key target was selected and included. Import the subcellular distribution information table into the Cytoscape software to draw the “subcellular-target” network diagram, as shown in Fig. 5 (B). The red V-shaped triangle node represents the key target, and the blue square node represents the subcellular structure. The key targets are mainly distributed in the extracellular (Degree = 6), plasma membrane (Degree = 5), endoplasmic reticulum (Degree = 3), nucleus (Degree = 3), which are also very important organelles.

Import the key targets into the DisGeNET database to obtain the protein information of each target. The results show that the key targets mainly involve transfer/carrier protein, signaling molecule, oxidoreductase, transferase and transcription factor. See Table 3 for details.

**Table 3:** Protein class of eight key targets for common pathways of Banxia Xiexin decoction in the treatment of depression and ulcerative colitis

**No.**	**Gene Symbol**	**Protein attribution information**	**No.**	**Gene Symbol**	**Protein attribution information**
**1**	ALB	transfer/carrier protein	**5**	PTGS2	oxidoreductase
**2**	IL6	None	**6**	MAPK1	transferase
**3**	VEGFA	signaling molecule	**7**	STAT3	transcription factor
**4**	TNF	signaling molecule	**8**	EGFR	None

## Discussion

As the basic principle of guiding the clinical diagnosis and treatment of diseases, the treatment method of “treating different diseases with the same treatment” of traditional Chinese medicine is also the essence of syndrome differentiation and treatment in traditional Chinese medicine. The explanation for the simultaneous treatment of different diseases is mainly attributed to the similar aetiology, pathogenesis, symptoms and disease location during the occurrence and development of different diseases. Therefore, different diseases can be cured with the same prescription, completely reflecting the advantages of traditional Chinese medicine in syndrome differentiation, holistic treatment and comprehensive treatment. Nowadays, the traditional Chinese medicine Banxia Xiexin Decoction is often used to treat ulcerative colitis [10, 11] and depression [26, 27], which have achieved better clinical effect. At the same time, it is widely concerned in the field of overall intervention of traditional Chinese medicine in the prevention and treatment of ulcerative colitis and depression. However, there is a lack of comprehensive and systematic understanding of the active ingredients, targets, molecular mechanisms, and tissue and subcellular distributions of the herbal medicines contained in the prescription in the formular. Therefore, based on the analysis of the biological network module of network pharmacology, a multi-level network containing the five components of “prescription-herbal medicine-component-target-disease” is constructed to observe the drug-disease interaction from an overall perspective. The potential regulatory mechanism of Banxia Xiexin decoction in the process of “treating different diseases with the same treatment” is further clarified.

According to the analysis of enriched biological processes and signalling pathways of common targets of the classical prescription used to treat two diseases, the GO biological processes, KEGG pathways, and the results of the biological network module analysis are overlapped and were enriched in the 5-HT synaptic signalling pathway, arachidonic acid (AA) metabolism, HIF-1 signaling pathway and NF-kappa B signalling pathway. Thus, different diseases are able to be treated with the same prescriptions. 5-hydroxytryptamine (5-HT), as an indole derivative, is mainly produced in brain tissue by tryptophan hydroxylase-2 (TPH2) and plays an important role. 95% of 5-HT is mainly produced in gastrointestinal tract of digestive system, and this process is mainly produced by further metabolism of tryptophan hydroxylase-1 (TPH1) [28]. 5-HT not only is expressed in neurons but also is involved in regulating the development, survival and apoptosis of neurons. 5-HT plays an important role in regulating the inflammatory response and in immunoregulation of the central nervous system. 5-HT is also a brain-gut peptide that is closely related to gastrointestinal activity and absorption. 5-HT has a variety of biological functions and plays a role in the physiological processes of gut sensation, movement and secretion, and in the pathological processes of gut inflammation and development [29, 30]. It is also found that 5-HT has a high content in cerebral cortex and synapses. It is not only used as an inhibitory neurotransmitter, but also related to synaptic transmission, neural signal transduction, neurotransmitter, emotion regulation, learning and memory regulation. At the same time, 5-HT also has a cross effect with arachidonic acid metabolism pathway. Another physiological function of 5-HT is regulation of arachidonic acid and eicosane metabolism. Once 5-HT is released from the presynaptic axon terminals, it will bind to the 5-HT2 receptor family to regulate the activity of phospholipase A2, which is responsible for the release of the phospholipid arachidonic acid. In addition, 5-HT synthesis and reuptake and arachidonic acid/prostaglandin metabolism are all related to the synaptic functions of 5-HT. [31] However, AA metabolism is closely related to the pathogenesis of ulcerative colitis. AA, a polyunsaturated fatty acid involved in the metabolism of multiple organs and systems in the body, is catalytically degraded by its rate-limiting enzyme COX-2 to produce a series of metabolites in the event of inflammation or an emergency. These metabolites play an important role in regulating the immune system of the body [32]. At the same time, related studies also show that the pathogenesis of ulcerative colitis is mainly affected by tryptophan metabolites, which also emphasizes the potential relevance between tryptophan metabolism and intestinal microbial regulation [33, 34]. Its potential pathogenesis is that tryptophan can be metabolized by intestinal bacteria into indole derivatives, such as indole-3-acetic acid (IAA), can also be metabolized by host cells into kynurenine (KYN) through indoleamine 2, 3-dioxygenase 1 (IDO 1) [35, 36]. Indole derivatives are aromatic hydrocarbon receptors (AHR) ligands to activate the production of IL-22 cytokines, that is, tryptophan catabolic metabolites produced mainly by the metabolism of microbial in the gastrointestinal tract activate the immune regulation of the body through AHR, and then maintain the intestinal homeostasis to promote the recovery of intestinal diseases [37, 38]. At the same time, 5-HT produced in the gut can indirectly affect the central serotonin pathway by regulating tryptophan metabolism through intestinal microorganisms, even though it can not pass through the blood-brain barrier [39]. However, tryptophan, as a precursor of 5-HT, is mainly metabolized through the kynurenine pathway, which produces a variety of biologically active metabolites and participates in a variety of physiological processes in the body. Related studies have shown that there is a close relationship between tryptophan-kynurenine metabolism pathway and depression in inflammatory state. When chronic stress or immune activation occurs, inflammatory factors induce the activation of indoleamine 2,3-dioxygenase, which becomes the first rate limiting enzyme of tryptophan degradation in kynurenine pathway. A large number of proinflammatory cytokines can also activate indoleamine 2,3-dioxygenase (IDO) and tryptophan 2,3-dioxygenase (TDO) through the inflammatory signal pathway, enhance the decomposition of tryptophan, reduce the level of peripheral tryptophan, thus reducing the amount of tryptophan supplied to the brain through the blood-brain barrier. At the same time, the presence of pro-inflammatory cytokines makes IDO1 in the brain also be overactivated and enhances the metabolism of tryptophan. The increased metabolism of tryptophan results in the decrease of tryptophan level in the brain, and ultimately results in the change of KYN and its downstream metabolites [40–42]. Therefore, it can be concluded that in the process of 5-HT and AA metabolism, classic formula can plays an important role in the treatment of different diseases by indirectly regulating the level of tryptophan metabolism. At the same time, it can also participate in the synergistic process of serotonin nervous system and brain-gut-axis, which further reflects the relationship between the nervous system and gastrointestinal system, and then effectively treats gastrointestinal dysfunction and depression symptoms, and fundamentally change the overall state of patient.

The targets of Banxia Xiexin decoction were also significantly enriched in the HIF-1 signaling pathway and NF-kappa B signalling pathway. Studies have confirmed that acute stress can promote animals to keep excited for a certain period of time, while long time in this state of tension will make the sympathetic nerve in the body in a continuous state of tension, the hypothalamic-pituitary-adrenocortical (HPA) axis over excitation will cause human body to be in a negative state, produce anxiety and despair, reduce the ability of learning and memory, and then the nervous system and immune system of the body will be in different levels of disorder, leading to the occurrence of various diseases, and serious mental diseases such as depression, which will become a serious threat to our own and social development [43]. However, hypoxia inducible factor (HIF) is a transcription activating factor, which is a heterodimer composed of HIF-αand HIF-β.HIF-αis its functional subunit, HIF-1α is greatly affected by oxygen concentration, which is of great significance to maintain homeostasis under hypoxia stress [44, 45]. As a neuroprotective factor, HIF-1α is widely expressed in neurons, glial cells and ependymal cells in the central nervous system. It can promote the transcription of downstream target genes through MAPK signal pathway and PI3K/Akt signal pathway to achieve the protective effect on tissues or systems. Meanwhile, HIF-1α can achieve the transcription by recognizing the hypoxia response element HRE in the nucleus. There is a hypoxia responsive element HRE binding to HIF-1 upstream of the transcription initiation site of vascular endothelial growth factor (VEGF). Under the condition of hypoxia, it can regulate the transcription of VEGF, promote angiogenesis and activate its downstream target genes such as inducible nitric oxide synthetase (iNOS) to increase blood flow and reduce the injury of ischemia and hypoxia. Erythropoietin (EPO) is also a downstream target gene of HIF-1α. There is HRE at the 3 ‘end of the gene which can be used for specific binding of HIF-1 α and regulate the transcription activation of EPO [46]. Some of resaerchs found that drugs or other treatments on rats with chronic cerebral ischemia may play a neuroprotective role by affecting the expression of HIF-1 α, which can be reduce the damage of nerve cells, and thus improve the learning and memory ability of rats with chronic cerebral ischemia [47–49]. Similarly, in mild cognitive impairment (MCI) subjects and early Alzheimer’s disease (AD) state, the expression of glycolytic factors increased, and HIF-1 mediated brain autoregulation may have early adaptation to oxidative and inflammatory stress in enhancing cerebral blood flow and antioxidant pathways, so as to reduce neuronal death [50]. Therefore, the increase of HIF-1 expression is a molecular adaptive response in the early stage of hypoxia. It can regulate the expression of many downstream target genes, not only for the nervous system, but also for digestive system diseases. A large number of studies have shown that nitric oxide (NO), as an important inflammatory mediators, participates in the occurrence and development of ulcerative colitis, and the expression of iNOS in colonic mucosa increases correspondingly [51]. The activation of HIF-1 α expression is related to inflammation. For example, lipopolysaccharide (LPS), a bacterial metabolite, can strongly induce the expression of HIF-1α in macrophages, thus playing an important role in LPS mediated inflammation. In the inflammatory model, knockout of HIF-1 gene can inhibit the ability of leukocytes (called macrophages) and neutrophils involved in the formation of inflammation to play a role in the hypoxic environment, thus preventing the emergence of inflammatory response, and the inflammatory exudation basically disappears [52]. Therefore, it can be said that HIF-1α is not only related to hypoxia, but also can be induced by IL-1βand TNF-α. Therefore, for ulcerative colitis with the existence of IL-1βand TNF-α, the production pathway of HIF-1α is more abundant, and the activation of HIF-1α can induce the production of IL-6, IL-8 and other inflammatory mediators such as iNOS and COX-2. It can be concluded that Banxia Xiexin Decoction can participate in the process of anti depression and prevention and treatment of ulcerative colitis by regulating cytokines and inflammatory mediators through HIF-1α.

Activated NF-kB, an important nuclear transcription factor in cells, participates in the mechanisms regulating the expression of various genes involved in the inflammatory response and immune response, and it regulate cell apoptosis, the stress response, synaptic plasticity and memory. In recent years, an imbalance in the intestinal flora has been reported to be closely related to the occurrence of ulcerative colitis. When ulcerative colitis occurs, it will activate the immune system in vivo and lead to the occurrence of systemic inflammation. T-lymphocytes will accumulate in the damaged intestinal mucosa and lead to a defect in intestinal barrier function. The immune system is activated by various microbial antigens after intestinal pathogens invade the submucosa. The activation of inflammatory cells leads to the inflammation of the intestinal mucosa. This process is mediated by Toll-like receptors, which activate the NF-kappa B signalling pathway, resulting in the release of the pro-inflammatory cytokines IL-1 and TNF-α. The inflammatory state caused by the abnormal immune function also plays a role in the pathogenesis of depression. The number of immune cells in patients with depression often changes the immune function of the body, disrupting the immune function of peripheral cells and increasing the levels of pro-inflammatory cytokines. Subsequently, the pro-inflammatory cytokines alter the local brain structure and neuroendocrine function. Therefore, Banxia Xiexin decoction may induce the expression of inflammatory cytokines and participate in the immune and inflammatory responses of patients with depression by activating the NF-kappa B signalling pathway to effectively improve the gastrointestinal inflammatory response and the effective regulation of the central nervous system [53, 54].

In this study, we used the tools of BioGPS, Genecards and DisGeNET to analyze the tissue distribution, subcellular distribution, and protein attribution. It can be seen from the results that in the treatment of depression and ulcerative colitis, the tissue distribution mainly involves the amygdala, pancreas and lung of brain, while the subcellular localization mainly includes myeloid cells, monocytes, cardiomyocytes, lymphoblasts and bronchial epithelial cells. Therefore, the prediction results not only provide a reference value for the clinical application of traditional Chinese medicine in the treatment of different diseases, but also provide guidance for the next animal and cell experimental verification stage of the research group, and also open up a new research idea for the traditional Chinese medicine to study the potential pharmacological mechanism of depression and ulcerative colitis from the perspective of brain, lung and pancreas.

## Conclusions

In summary, this paper studies the common mechanism of the treatment of ulcerative colitis and depression using a network pharmacological biological module analysis by investigating biological processes, signalling pathways, tissue distribution, subcellular distribution, protein attributes, etc. Banxia Xiexin decoction not only directly participated in drug and lipid metabolism but also exerted the effect of “treating different diseases with the same treatment” by comprehensively regulating the functions of the nervous system, immune system, digestive system and other systems of the body. In addition, from the perspective of integration, we also explored the efficacy of this herbal medicine through multiple channels and effects. The results were consistent with the process of disease induction by multiple factors and interactions, and a certain correlation was observed between the key signalling pathways that work synergistically. The traditional concept of “treating different diseases with the same treatment: of traditional Chinese medicine is integrated with the research technology of modern network pharmacology to not only take full advantage of traditional Chinese medicine in overall treatment and comprehensive prevention and treatment but also systematically analyse the mechanism underlying the efficacy of ancient traditional Chinese medicine prescriptions using of modern visualization technology. Our study provides a new area of research for exploring the mechanism of synergism in the treatment of different diseases, provides a reliable reference value for the subsequent experimental verification of our findings, and ensures that the overall research results are highly credible and persuasive.

## Data Availability

The data used to support this study can be obtained from the corresponding authors, according to the requirements of the journal. The data supporting the relevant research conclusions are freely available.

## Abbreviations

QED: The quantitative estimate of drug-likeness
OB: Oral bioavailability
DL: Drug likeness
PPI: Protein-protein interaction network
GO: Gene ontology
KEGG: The Kyoto encyclopedia of genes and genomes
MTHFR: Methylenetetrahydrofolate reductase
TLR4: Toll-like receptor 4
GSTP1: Glutathione S-transferase P1
5-HT: 5-hydroxytryptamine
AA: Arachidonic acid
COX-2: Cyclooxygenase-2
GSH: Glutathione synthetase
HPA: Hypothalamic pituitary adrenocortical
HIF: Hypoxia inducible factor
VEGF: Vascular endothelial growth factor
MCI: Mild cognitive impairment
AD: Alzheimer’s disease
